# Older Age Predicts Better Psychological Well-Being Even After Experiencing a Disaster

**DOI:** 10.1093/geroni/igaf122.2281

**Published:** 2025-12-31

**Authors:** Esha Azhar, JoNell Strough

**Affiliations:** West Virginia University, Morgantown, West Virginia, United States; West Virginia University, Morgantown, West Virginia, United States

## Abstract

Climate change is associated with the increased prevalence of extreme weather and disasters, which can pose negative consequences for well-being. Yet, post-disaster benefits of aging for well-being have been found, such that the benefits were more pronounced among those who had experienced a disaster versus not (Strough et al., 2023). Aiming to replicate this finding, adult age differences in psychological well-being were compared among people who had experienced a disaster versus not in 2023. An exploratory, secondary analysis of data from the Understanding America Study (UAS), a national US panel, was conducted. Data from two surveys, UAS 588 (N = 10,193) and UAS 592 (N = 9,591), containing panelists’ self-reported experience with disasters (e.g. wildfires, hurricanes, earthquakes, etc.), and self-reported psychological well-being in the last 14 days (e.g. “Feeling nervous, anxious, or on edge”, “Feeling down, depressed, or hopeless”, etc.) respectively, were merged and analyzed. A multiple linear regression analysis predicting psychological well-being was significant, F(2,9202) = 328.37, R² = .10, p < .001. Significant predictors of worse psychological well-being were younger age, disaster exposure, annual household income below $75k, education less than a bachelor’s degree, and being female. A significant age by disaster interaction indicated that among those who had experienced a disaster, the association between older age and better psychological well-being was even stronger compared to those who had not experienced a disaster. We find that older adults possess better psychological well-being overall and post-disaster, indicating this group’s resilience and ability to cope with adverse circumstances.

